# Streamlined concomitant pulse field ablation–based pulmonary vein isolation and left atrial appendage occlusion via a single venous access approach: a case report

**DOI:** 10.1093/ehjcr/ytaf350

**Published:** 2025-07-26

**Authors:** Christian-H Heeger, Henning Rolfes, Lena Böttcher, Felix Meincke, Martin W Bergmann

**Affiliations:** Department of Rhythmology, Cardiology and Internal Medicine, Asklepios Klinik Altona, Paul-Ehrlich-Straße 1, Hamburg 22763, Germany; German Center for Cardiovascular Research (DZHK), Partner Site Hamburg/Kiel/Lübeck, Lübeck, Germany; Department of Rhythmology, University Heart Center Lübeck, University Hospital Schleswig-Holstein, Ratzeburger Allee 160, 23562 Lübeck, Germany; Department of Rhythmology, Cardiology and Internal Medicine, Asklepios Klinik Altona, Paul-Ehrlich-Straße 1, Hamburg 22763, Germany; Department of Rhythmology, Cardiology and Internal Medicine, Asklepios Klinik Altona, Paul-Ehrlich-Straße 1, Hamburg 22763, Germany; Department of Rhythmology, Cardiology and Internal Medicine, Asklepios Klinik Altona, Paul-Ehrlich-Straße 1, Hamburg 22763, Germany; Department of Rhythmology, Cardiology and Internal Medicine, Asklepios Klinik Altona, Paul-Ehrlich-Straße 1, Hamburg 22763, Germany

**Keywords:** Pulsed field ablation, Pulmonary vein isolation, Left atrial appendage closure, Case report

## Abstract

**Background:**

Pulsed field ablation (PFA) is a novel non-thermal cardiac ablation method utilizing irreversible electroporation which has been introduced especially for treatment of atrial fibrillation (AF) by pulmonary vein isolation (PVI). Interventional left atrial appendage closure (LAAC) is an alternative to oral anticoagulation (OAC) in patients with non-valvular AF and high stroke risk who are ineligible for OAC. A concomitant PVI and LAAC might be beneficial for patients.

**Case summary:**

In an 82-year-old male patient with symptomatic persistent AF, CHA_2_DS_2-_VA Score of 4 and HASBLED Score of 3 due to previous gastrointestinal bleeding were scheduled for a concomitant PVI + LAAC procedure. For minimum risk, a streamlined approach utilizing a single femoral vein puncture in combination with a suture-based closure system (Perclose Prostyle, Abbott) was performed. The transoesophageal echocardiography (TOE) LAA landing zone measurements were achieved directly prior ablation. Pulmonary vein isolation was performed with pentaspline PFA catheter (FARAPULSE). Although a swelling of the left atrial ridge was observed, a 24 mm WATCHMAN FLX device was successfully implanted. The patient was mobilized after 2 h and discharged on the next day. After 2 months on OAC, TOE found no gaps or leakage of the LAAC device and OAC was switched to acetylsalicylic acid monotherapy.

**Discussion:**

A streamlined concomitant PFA-based PVI and LAAC procedure utilizing FARAPULSE and WATCHMAN FLX devices seems to be feasible and safe. Swelling of the ridge after PVI was observed; however, the sizing measurements have been performed prior PVI, and the LAAC procedure was successful with no evidence for gaps or leakage. A concomitant approach might be a suitable option for selected patients.

Learning points

- A streamlined concomitant pulsed field ablation–based pulmonary vein isolation (PVI) and left atrial appendage closure (LAAC) procedure seems to be feasible and safe.
- Since swelling of the ridge could be observed and might be an issue for the long-term LAAC success, the sizing measurements should be performed prior PVI.

## Introduction

Atrial fibrillation (AF) catheter ablation by pulmonary vein isolation (PVI) and left atrial appendage (LAA) closure (LAAC) for stroke prevention are increasingly performed as individual procedures. However, there are several studies which investigated a combined concomitant approach of performing a thermal ablation–based PVI and LAAC in one single procedure and safety and efficacy have been shown.^1–4^ The recently published OPTION trial showed that PVI and LAAC (either sequential or concomitant) was non-inferior to oral anticoagulation (OAC) with respect to a composite of death from any cause, stroke, or systemic embolism at 36 months.^5^ However, only thermal-based PVI has been utilized and only 40.8% of patients have been treated concomitant. The novel ablation modality of pulsed field ablation (PFA) has significantly reduced procedure duration and potentially increased safety due a specific ablation of cardiac tissue with sparing smooth muscle cells and nerval cells.^6^ With currently >220.000 treated patients, most experience is currently available for the FARAPULSE PFA system (Boston Scientific, Plymouth, MN, USA). The MANIFEST 17K registry showed an excellent safety and efficacy profile of this novel ablation technology.^7^ In patients with non-valvular AF, at high stroke risk, and who are ineligible for long-term OAC, LAAC could be an alternative to OAC and safety and efficacy have been shown in several studies.^8,9^ While PVI and LAAC are both conducted in the left atrium (LA) and share the same access route, a combined concomitant approach might be beneficial for patients, but is not conventionally practiced. Due to significantly shorter procedures times and a high safety profile, PFA-based catheter ablation may be advantageous for the combined approach of PVI and LAAC. Further potential benefits are the reduction of peri-procedural complications. Furthermore, economic factors could be beneficial due to only one concomitant procedure with one hospital stay instead of two procedures with two hospital stays. Here, we are presenting a case of concomitant PFA-based PVI and LAAC utilizing the WATCHMAN FLX LAAC system (Boston Scientific).

## Summary figure

**Figure:**
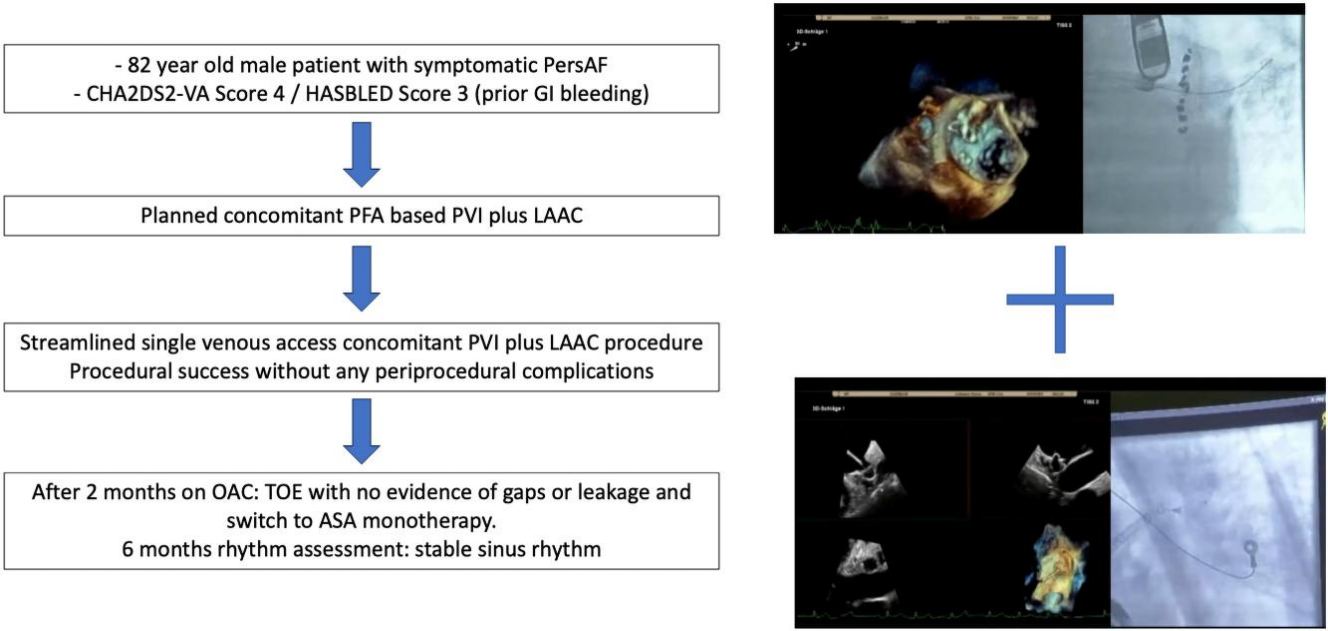


## Case presentation

**Figure 1:**
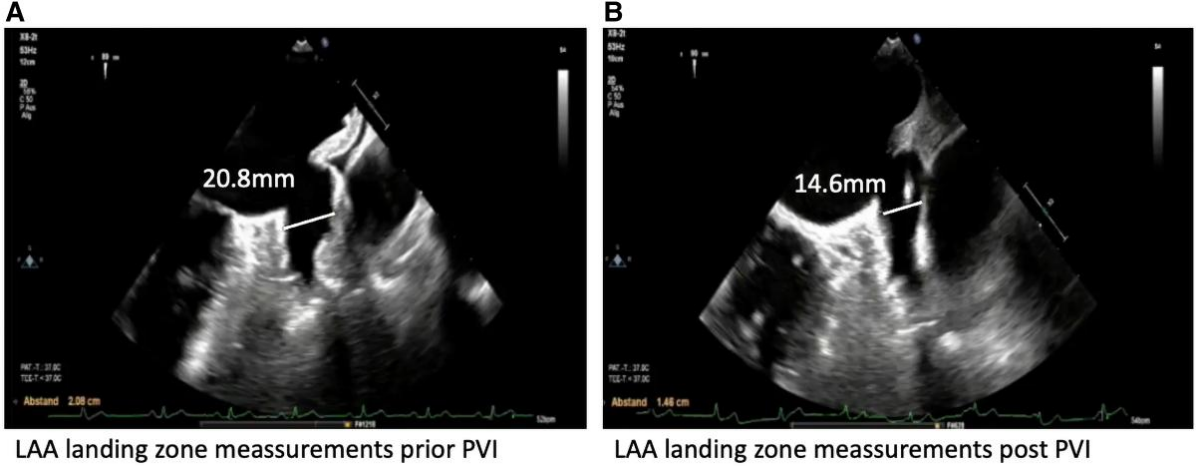
Left atrial appendage landing zone measurements prior and post pulmonary vein isolation. (*A*) Prior pulmonary vein isolation. (*B*) Post pulmonary vein isolation. The maximal diameter of the left atrial appendage landing zone was 20.8 mm prior pulmonary vein isolation and 14.6 mm after pulmonary vein isolation.

**Figure 2:**
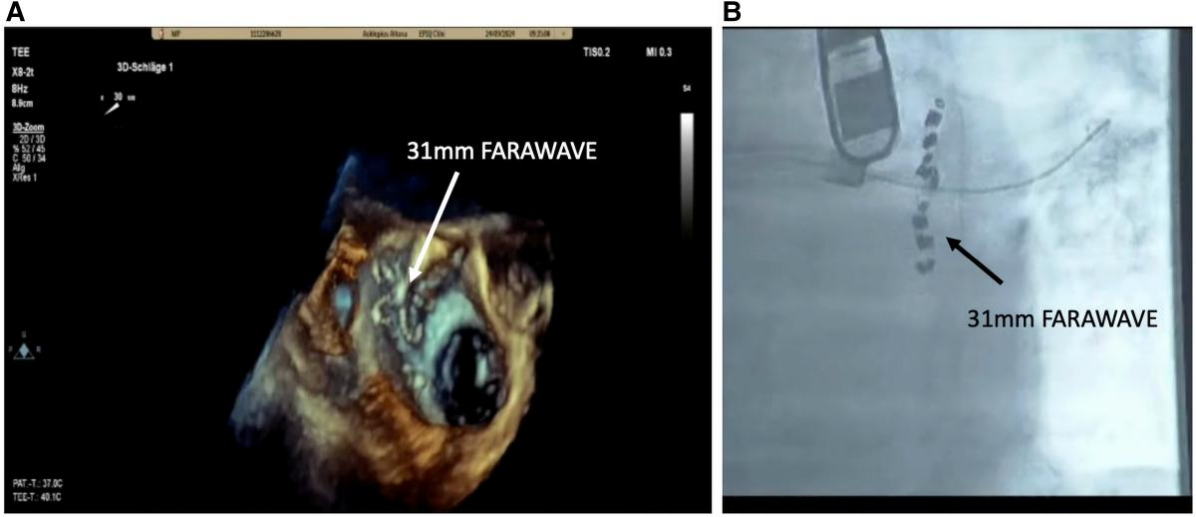
Pulsed field ablation–based pulmonary vein isolation utilizing the FARAPULSE system. 3D transoesophageal echocardiography image (*A*) and fluoroscopy image (*B*) showing the Farawave catheter in the flower position at the ostium of the left inferior pulmonary vein.

**Figure 3:**
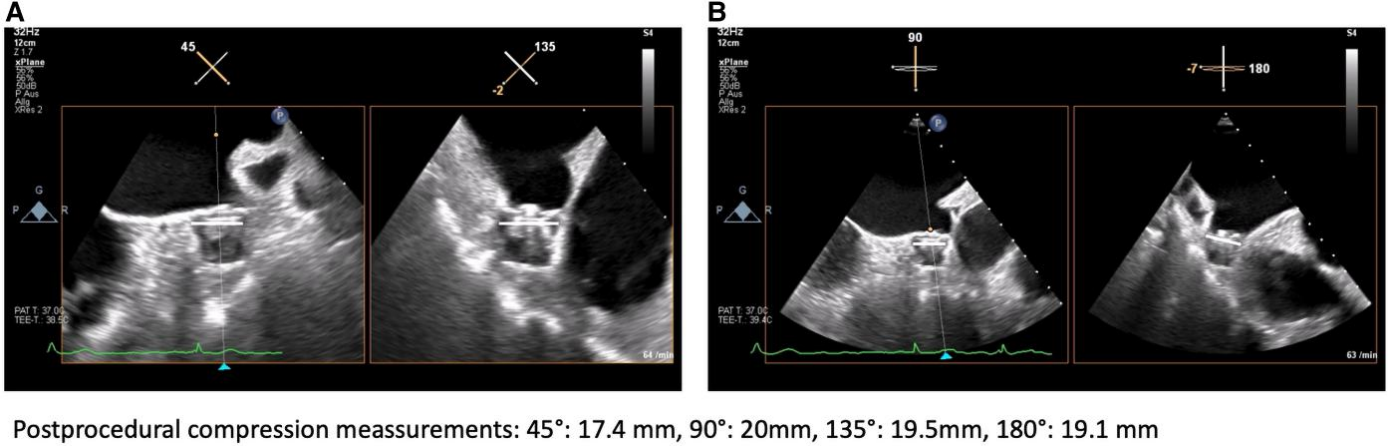
Post left atrial appendage closure implantation measurements: (*A*) 45° and 135° and (*B*) 90° and 180°.

An 82-year-old male patient with symptomatic persistent AF, intolerance to beta-blockers, and contraindication to amiodarone due to history of hyperthyroidism presented for AF catheter ablation. A preserved left ventricular ejection fraction with mild bi-atrial dilatation was observed. The CHA_2_DS_2-_VA Score was 4. After an upper gastrointestinal bleeding, the HASBLED Score was 3. According to latest guidelines with symptomatic persistent AF, PVI was recommended by Class I; furthermore, an indication for LAAC was recommended by Class IIb.^10^ After informed consent, the patient was scheduled for a concomitant PFA-based PVI and LAAC by WATCHMAN FLX. Single femoral vein vascular access was achieved utilizing ultrasound-guided puncture of the right femoral vein (1 × 8 F). Additionally, a venous closure system (Perclose Prostyle, Abbott Vascular) was prepared.^11^ No coronary sinus catheter was utilized. A single transseptal puncture (TSP) was performed under fluoroscopic and transoesophageal echocardiography (TOE) guidance using a modified Brockenbrough technique via a SL1 sheath. After TSP, heparin boluses were administered targeting an activated clotting time of >300 s. Angiography of pulmonary veins was performed utilizing contrast medium directly via the SL1 sheath. No pre- and post-3D mapping has been performed. The measurements of the LAA landing zone were performed prior PVI with a maximal diameter of 20.8 mm (at 90°) (*Figure 1*). The SL1 sheath was exchanged for the Faradrive sheath (15.8 F), and the 31 mm Faradrive ablation catheter was advanced to the LA. Prior to the first pulse delivery, 1 mg atropine was given intravenously to avoid vagal reaction such as sinus arrest or intermittent atrioventricular block. Detailed description of the ablation protocol has been described before.^12,13^ In brief, eight pulse trains (2 kV/2.5 s, bipolar, biphasic, 4 × basket/flower configuration each) were delivered to each PV starting on the left-sided veins. Pulmonary vein isolation was determined by checking for absence of electrograms on the ablation catheter following ablation (*Figure 2*). Following PVI, the Faradrive sheath was exchanged to a 14 F WATCHMAN sheath. An angiographic and echocardiographic visualization of LAA landing zone was repeated with a maximal LAA diameter of 14.6 mm (90°). We assumed a temporary oedema of the left atrial (LA) ridge after PVI. Therefore, we decided to take a 24 mm WATCHMAN FLX device due to manufacturer’s recommendations with measurement before PVI (20.8 mm). Under TOE guiding, the WATCHMAN FLX device was positioned to the LAA and was released without any leakage and good compression (20 mm at 90°) (*Figure 3*). The procedure time was 65 min with a fluoroscopy time of 11.3 min (DAP 3575.66 cGy*cm^2^). Despite the fact that an upsizing from an 8 F SL1 to a 15.8 F (Faradrive) and downsizing to 14 F (WATCHMAN sheath) have been performed throughout the procedure, no oozing of the puncture site has been observed. The vascular closure device (Prostyle) was deployed, and a 2 h pressure bandage was used to prevent femoral bleeding. The patient was mobilized and discharged after exclusion of pericardial effusion on the day. The patient was treated with OAC (apixaban, 5 mg twice daily for 2 months). Control TOE after 2 months showed a complete sealed LAA with no evidence of gaps of leakage, and OAC was changed to acetylsalicylic acid 100 mg monotherapy. At 6 months of follow-up, the patient showed no evidence of stroke or bleeding and stable sinus rhythm without antiarrhythmic drug therapy.

## Discussion

Here, we are presenting a case of concomitant PFA-based PVI and LAAC utilizing a single access approach. The advantages of a single combined procedure in patients with an indication for both strategies are the potential reduction of peri-procedural complications and higher convenience for the patients, but also economic factors are playing a role in this topic. Two months after successful PVI and LAAC, the OAC therapy could be terminated which might be beneficial for the patients, especially for those with a higher bleeding risk.^10^ We showed that a concomitant PFA-based PVI and LAAC is feasible and potentially safe. However, due to the detected oedema of the LA-LAA ridge after ablation, the LAA landing zone diameter should be measured before PVI to avoid undersizing the LAAC device. Swelling of the LA ridge after PFA-based PVI was previously shown by Gaggiotti *et al*.,^14^ and a 27 mm WATCHMAN FLX device was successfully implanted. At follow-up after 6 weeks, the swelling was resolved and the compression was stable, yet a slightly titled device position was observed which was nevertheless stable and showed no leakage. Since a similar oedema creation of the LA-LAA ridge was observed in our case, the sizing of the landing zone should be done prior PVI. Although a recent case report on patients with LAAC devices showed impairment of PFA-based PVI,^15^ another option could be to change the order of the concomitant procedure starting with the LAAC procedure followed by PVI. Recently, a retrospective registry with *n* = 36 patients showed that combined FARAPULSE-based PFA and WATCHMAN FLX-based LAAC were feasible. In this study, a mean of 69.4% increase in pulmonary ridge thickness immediately following PFA was observed.^16^ However, no large-scale studies are available today, and more data are necessary to achieve.

Our case shows feasibility of a streamlined concomitant PFA-based PVI and LAAC with a WATCHMAN FLX device. In comparison to the reported findings by Gaggiotti *et al*.^14^ where a slightly tilted device position was detected at follow-up, no device impairment or position deviation was observed. The workflow described in this report is streamlined single ultrasound-guided venous puncture, single catheter approach with utilization of a venous closure system. It might be suitable approach for concomitant PVI and LAAC procedures by limiting the puncture sights, material, and costs. No pre-procedural CT scan has been performed due to the local institute’s workflow. It could be a suitable option for selected patients with indication for PVI and LAAC to treat AF and prevent stroke and bleeding with one single procedure. Since an oedema of the ridge was observed after ablation, this fact should be considered in concomitant procedures concerning the LAAC implantation strategy. By performing the landing zone measurement prior PVI, no post-procedural peri-device leakages, dislodgement, or impairment of the LAAC device was found. Currently, there are several unanswered questions including patient selection, reimbursement, device selection, and order of the procedure. Further studies with larger patient populations regarding concomitant LAAC and PVI are therefore necessary and planned [COCONUT (COnCOmitaNt Pulse Field Ablation Based pUlmonary Vein Isolation and lefT Atrial Appendage Closure) registry].

## Patient’s perspective

The advantages of a single combined procedure in patients with an indication for PVI and LAAC are the potential reduction of peri-procedural complications and higher convenience by reducing the treatment to just one hospital stay. Especially for patients with a high bleeding risk 2 months after successful PVI and LAAC, the OAC therapy could be terminated and bleeding episode could potentially be avoided.

## Data Availability

The data underlying this article will be shared on reasonable request to the corresponding author.

## Lead author biography

Christian-Hendrik Heeger is an electrophysiologist and the head of the Department of Rhythmology at the Asklepios Klinik Altona in Hamburg, Germany.

**Consent:** The authors confirm that written consent for submission and publication of this case report including images and associated text has been obtained from the patient, in line with the COPE guidance.

**Funding:** None declared.
